# Clinical, Functional, and Ultrasonographic Assessment of the Shoulder–Elbow Complex in Padel Players with Lateral Elbow Tendinopathy: A Pilot Observational Study

**DOI:** 10.3390/sports14080357

**Published:** 2026-08-18

**Authors:** Luis Morales-Rodríguez-Parets, Maria Gabriela Cortiguera, Blanca De-la-Cruz-Torres

**Affiliations:** 1 Department of Physiotherapy, Spanish Surfing Federation, 39005 Santander, Spain; luis@olympicsantander.es; 2 Olympic Physiotherapy and Sports Medicine Centre, 39005 Santander, Spain; mcortiguera@live.com; 3 Department of Physiotherapy, University of Seville, c/Avicena s/n, 41009 Seville, Spain

**Keywords:** tennis elbow, range of motion, shoulder joint, athletes, biomechanics

## Abstract

Introduction: Lateral elbow pain (LEP) is traditionally considered a local tendinopathy; however, emerging evidence suggests that proximal shoulder function and kinetic chain adaptations may contribute to its development in overhead and racquet sport athletes such as padel players. This study compared shoulder strength, neuromuscular activation, elbow tendon morphology, and grip strength between padel players with lateral elbow pain and healthy controls, and between pain and non-pain sides within the symptomatic group. Methods: A pilot study was conducted in padel players with unilateral LEP and matched healthy controls. Assessments included isometric shoulder internal and external rotator (IR/ER) strength, glenohumeral rotational range of motion, electromyographic activity (infraspinatus/deltoid posterior and upper trapezius/lower trapezius ratios), grip strength, and ultrasound evaluation of infraspinatus and common extensor tendon thickness. Results: No significant differences were observed between groups or sides in shoulder strength, IR/ER ratios, electromyographic activity, or grip strength (all *p* > 0.05). The symptomatic group showed greater infraspinatus muscle thickness than controls. Within the symptomatic group, the pain side demonstrated reduced glenohumeral IR (*p* = 0.04) and increased common extensor tendon thickness (*p* = 0.02–0.05). Conclusions: Padel players with LEP demonstrated largely preserved neuromuscular and strength profiles. However, reduced shoulder IR and increased tendon thickness suggest proximal mobility alterations and local tendon adaptations, supporting a kinetic chain interpretation of lateral elbow pain.

## 1. Introduction

Padel has experienced rapid growth over the last decade, becoming one of the fastest-growing racket sports worldwide [1]. Its repetitive high-velocity strokes, overhead actions, rapid accelerations and decelerations, and asymmetrical loading expose the upper limb to substantial mechanical stress, making the shoulder and elbow particularly susceptible to injury [2,3]. Epidemiological studies report injury rates ranging from 3 to 8 injuries per 1000 h of play, with the upper extremity being one of the most frequently affected regions [2]. Among these injuries, lateral elbow pain (LEP), or lateral elbow tendinopathy (LET), is one of the most common overuse disorders in both recreational and competitive players, primarily resulting from the repetitive execution of backhand strokes, volleys, and rapid wrist and forearm acceleration–deceleration movements [3,4].

Current evidence conceptualizes LET not merely as an isolated tendon disorder but as a complex neuromusculoskeletal condition involving structural, sensorimotor, and kinetic-chain impairments [5]. Coombes et al. [5] proposed an integrative model in which tendon pathology coexists with altered pain processing, motor dysfunction, and deficits in upper-limb load distribution. In this context, shoulder mechanics, cervical dysfunction, and deficits in scapular stabilization may modify force transmission along the upper extremity and contribute to persistent lateral elbow symptoms [6].

Scientific evidence suggests that lateral elbow tendinopathy should not be considered an isolated local tendon disorder, but rather a condition integrated within the entire upper-limb kinetic chain and cervicoscapular complex. Several investigations have demonstrated associations between lateral elbow pain and glenohumeral mobility deficits, reduced shoulder external rotation strength, scapular dyskinesis, and impaired proximal stability [6,7,8]. Kibler and Sciascia [7] proposed that alterations in scapular control may impair efficient force transfer during overhead movements, consequently increasing distal mechanical stress at the elbow joint. Likewise, Myers et al. [8] demonstrated that glenohumeral internal rotation deficits and posterior shoulder tightness can significantly alter upper-limb kinetic mechanics in overhead athletes.

Recent investigations have also emphasized the importance of ultrasound imaging and tendon structural assessment in the evaluation of lateral elbow disorders [9,10,11]. Tendon thickening, neovascularization, hypoechogenicity, and cortical irregularities have been reported in symptomatic populations, although imaging findings do not always correlate directly with pain intensity or functional disability [10]. This reinforces the multifactorial nature of LET and the need for comprehensive clinical assessment integrating biomechanical, functional, and imaging variables [10].

Despite the growing scientific interest in LET, there remains limited evidence regarding the functional and structural differences between symptomatic and asymptomatic players, as well as the bilateral adaptations associated with limb dominance in high-level athletes. Therefore, the aim of the present study was to analyze the clinical, functional, and ultrasonographic characteristics of the shoulder and lateral elbow complex in padel players, comparing players with different clinical statuses and pain versus non-pain upper-limb adaptations.

## 2. Materials and Methods

### 2.1. Design

A preliminary case–control study was conducted to estimate the sample size and assess the feasibility of a subsequent large-scale clinical trial. Thirty participants were recruited, and all recruitment procedures were performed at a private sports center.

### 2.2. Participants

**Figure 1:**
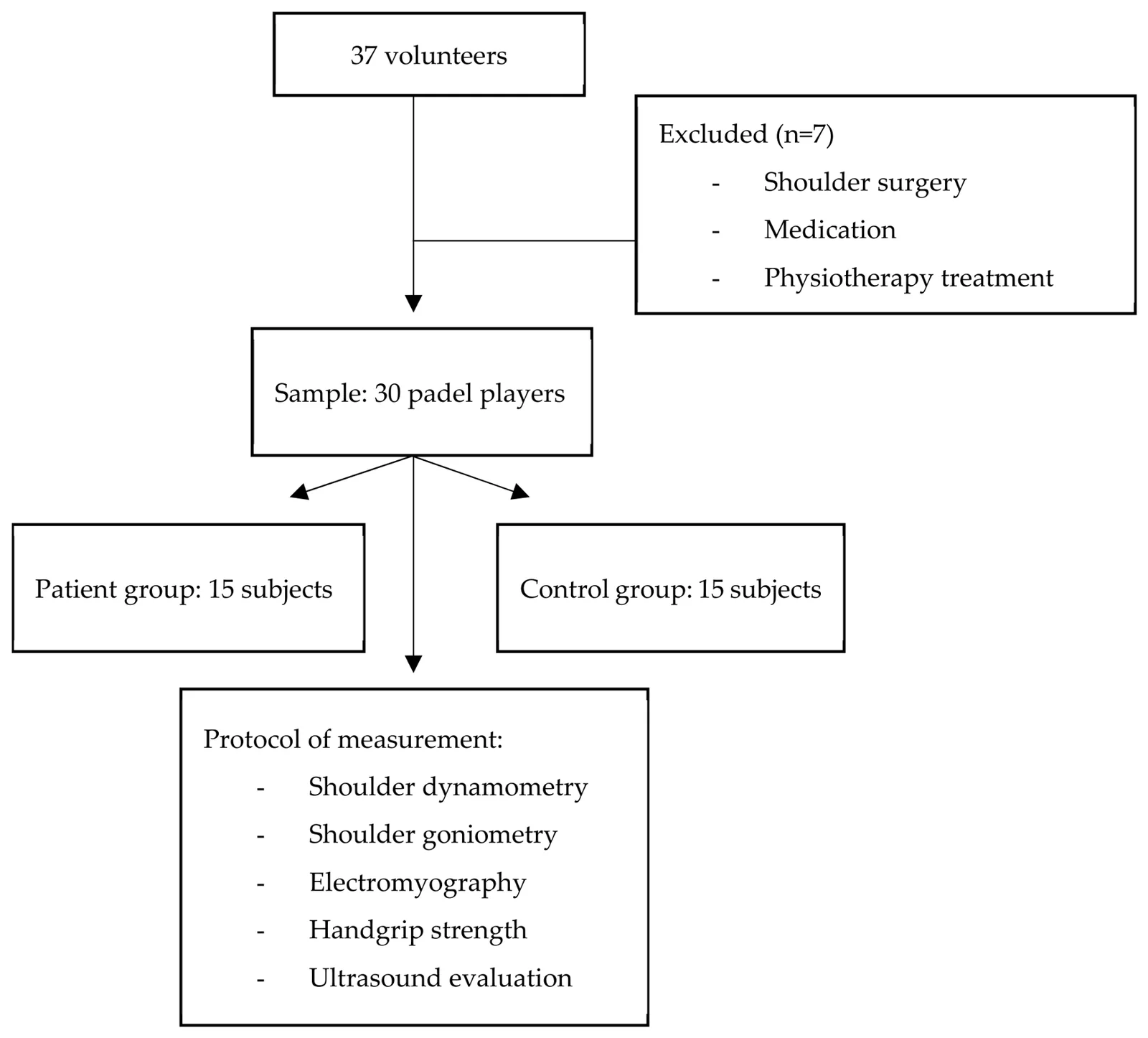
Flow diagram of patient recruitment and retention.

Thirty padel players (5 female, 25 male) were included in the study: the patient group consisted of 15 subjects (3 female, 12 male) presenting unilateral pain localized to the lateral aspect of the elbow, and in all participants, the dominant side coincidentally corresponded to the symptomatic side; and the control group was composed of 15 healthy subjects (2 female, 13 male). All players were actively competing at the time of data collection. As previously described in detail [12], inclusion criteria for the patient group comprised the presence of elbow pain persisting for at least three months, a pain insensitive ≥ 3 points on the visual analog scale (VAS), and positive Cozen, Mills, and Maudsley tests [13,14,15]. Exclusion criteria included radicular pain, previous upper-limb surgery or acute trauma, bilateral symptoms, electrophysiological signs suggestive of other peripheral nerve involvement, current pharmacological treatment, or concurrent physiotherapeutic interventions (including massage, therapeutic exercise, or electrotherapy) before or during the study. Participants who engaged in exercise therapy during the intervention period were also excluded. Seven participants were excluded based on the predefined exclusion criteria. Figure 1 shows the flow diagram of patient recruitment and retention.

As this was a pilot study, an a priori sample size calculation was not performed. Pilot studies are primarily intended to assess the feasibility of the study protocol and to provide preliminary estimates of outcome variability and effect sizes, which can subsequently be used to calculate the required sample size for a fully powered confirmatory study. Therefore, a convenience sample was considered appropriate for the aims of the present investigation [16]. Table 1 shows the descriptive characteristics of the sample.

**Table 1:** Descriptive characteristics of the sample. Data are presented as mean ± standard deviation.

	Patient Group	Control Group	*p* Value
Age (years)	36.70 ± 6.80	33.60 ± 9.50	0.32
Weight (kg)	70.40 ± 9.70	70.00 ± 11.30	0.92
Height (cm)	172.50 ± 9.60	169.10 ± 7.20	0.28
Pain (VAS)	4.53 ± 1.25	0.00 ± 0.00	-
Pain duration (months)	16.13 ± 10.40	0.00 ± 0.00	-
Years competing	6.90 ± 6.20	8.70 ± 7.50	0.45
Training volume (hours/week)	13.10 ± 11.20	9.60 ± 8.50	0.33
Previous injuries (dominant/painful side)	1.20 ± 1.00	0.80 ± 1.00	0.30

Abbreviation: visual analog scale, VAS. Note: For VAS and pain duration, the independent samples *t*-test could not be performed due to zero variance after grouping by study group.

### 2.3. Ethical Considerations

The study was approved by the Ethics Committee of the Virgen Macarena and Virgen del Rocio University Hospitals (protocol code 1193-N-17) and conducted in accordance with the principles of the Declaration of Helsinki. All participants provided written informed consent prior to their inclusion in the study.

### 2.4. Clinical Assessment

**Figure 2:**
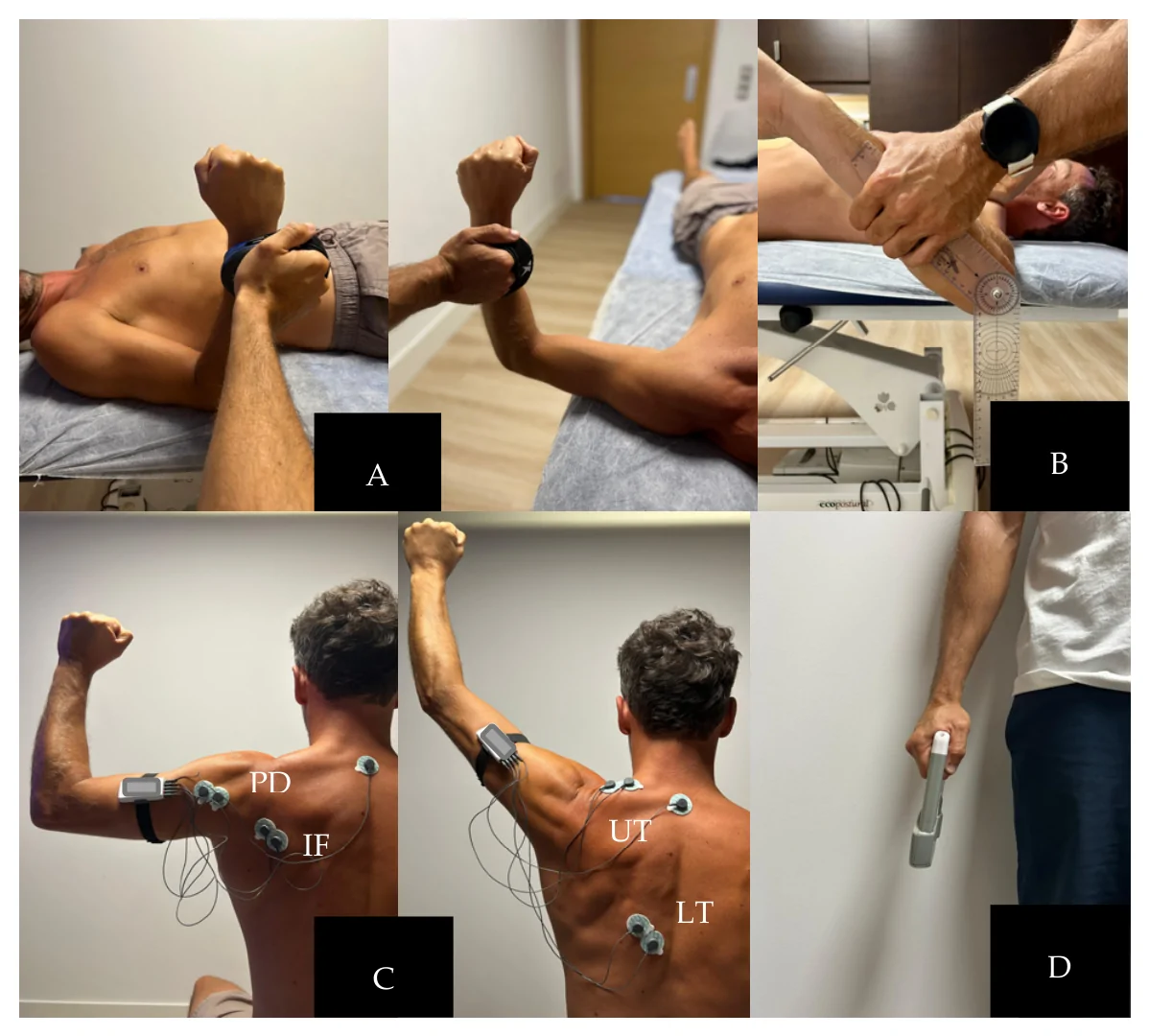
(**A**) Shoulder dynamometry at 0° and 90 of abduction; (**B**) passive glenohumeral internal and external rotation using a universal goniometer; (**C**) electromyographic activity of the infraspinatus/posterior deltoid (**A**) during external rotation; and upper trapezius/lower trapezius (**B**) during shoulder abduction. IF, infraspinatus; PD, posterior deltoid; UT, upper trapezius; LT, lower trapezius; (**D**) handgrip strength measurement.

Shoulder Dynamometry. Shoulder internal rotation (IR) and external rotation (ER) strength were assessed using a handheld dynamometer (ActivForce 2 handheld dynamometer, Sunnyvale, CA, USA) under two testing conditions: with the shoulder positioned at 0° and 90° of abduction (Figure 2A) [17]. All measurements were performed bilaterally by the same experienced physiotherapist following a standardized protocol [18]. For both testing conditions, participants were positioned in supine decubitus. During the assessment at 0° of shoulder abduction, the shoulder was maintained in neutral abduction and rotation, with the elbow flexed to 90° and the forearm in neutral position. During the assessment at 90° of shoulder abduction, the shoulder was positioned at 90° abduction in the scapular plane with the elbow flexed to 90°, maintaining neutral forearm alignment throughout testing. The physiotherapist stabilized the participant proximally to minimize compensatory trunk and scapular movements during force production. The handheld dynamometer was positioned at the distal forearm, proximal to the ulnar styloid process. Participants performed maximal voluntary isometric contractions against the dynamometer for approximately 5 s with standardized verbal encouragement. Three repetitions were performed for each movement and limb, with a 30 s rest interval between attempts. The mean value of the three measurements was calculated and used for statistical analysis. The following variables were analyzed bilaterally at both 0° and 90° of shoulder abduction: IR strength, ER strength, IR/ER ratio and Inter-limb asymmetry percentage for IR and ER strength. The IR/ER ratio was calculated as the quotient between IR and ER strength values, while inter-limb asymmetry was calculated as the percentage difference between dominant and non-dominant limbs: Asymmetry (%) = [(Dominant − Non dominant)/Dominant] × 100.

Shoulder Goniometry. Passive glenohumeral IR and ER range of motion (ROM) were assessed bilaterally using a universal goniometer [19,20]. Participants were positioned supine with the shoulder abducted to 90° and the elbow flexed to 90° [17]. The scapula was manually stabilized by the physiotherapist to minimize compensatory scapulothoracic movement during testing. For both IR and ER measurements, the fulcrum of the goniometer was aligned with the olecranon process of the ulna. The stationary arm was positioned perpendicular to the floor, while the movable arm was aligned with the ulna toward the ulnar styloid process. During internal rotation assessment, the examiner passively moved the forearm anteriorly toward the examination table until the first point of scapular compensation or firm end-feel was detected. For external rotation assessment, the forearm was moved posteriorly following the same criterion. Three measurements were obtained for each movement, and the mean value was used for analysis.

Total range of motion (TROM) was calculated as the sum of IR-ROM and ER-ROM: Total arc of motion = IR + ER.

Glenohumeral internal rotation deficit (GIRD) was calculated as the difference in IR-ROM between the pain and non-dominant shoulders: GIRD = IR dominant-IR non-dominant. Currently, GIRD can be defined by a loss of >20° of IR compared to the contralateral shoulder [17,21].

Electromyography. Surface electromyography (mDurance^®^, Granada, Spain) system was used to assess muscle activation patterns of the shoulder stabilizing musculature during standardized tasks. Bipolar Ag/AgCl surface electrodes were placed following SENIAM recommendations after skin preparation, including shaving (if necessary), abrasion, and cleaning with alcohol to reduce skin impedance [22]. Electrode placement was performed parallel to muscle fiber orientation with an inter-electrode distance of 20 mm. For the infraspinatus/posterior deltoid ratio (the IF/PD ratio) at 90° of shoulder abduction (Figure 2C), electrodes were positioned over the IF muscle belly below the scapular spine and over the PD approximately 2 cm inferior and lateral to the posterolateral acromion. Participants were seated and performed resisted external rotation tasks with the shoulder positioned at 90° abduction and elbow flexed to 90°, completing 5 repetitions using 2 kg dumbbell. The IF/PD ratio was calculated by dividing normalized IF activity by PD activity. For the upper trapezius/lower trapezius ratio (UT/LT ratio) during shoulder abduction (Figure 2B,C), electrodes were placed over the upper trapezius midway between the acromion and C7 spinous process, and over the lower trapezius approximately two-thirds of the distance between the scapular spine and T8 vertebra. Participants were seated and performed active shoulder abduction in the scapular plane while holding a 2 kg dumbbell. The UT/LT ratio was calculated as the normalized upper trapezius activity divided by lower trapezius activity. The following electromyographic variables were obtained bilaterally: IF/PD and UT/LT electromyographic ratios during shoulder abduction tasks in pain and non-pain limbs.

Handgrip Strength. This variable was measured using a calibrated handheld dynamometer (CAMRY Electronic Co., Ltd., Zhongshan, China) (Figure 2D). Participants were positioned seated with the shoulder adducted and neutrally rotated, elbow flexed to 90°, forearm in neutral position, and wrist between 0° and 30° extension, following the recommendations of the American Society of Hand Therapists [23]. Participants performed three maximal voluntary contractions for each hand, maintaining each contraction for approximately 3–5 s with standardized verbal encouragement. A 30 s rest interval was allowed between trials. The highest value obtained for each side was used for statistical analysis. The following variables were analyzed: dominant handgrip, non-dominant handgrip and handgrip asymmetry (%). Handgrip asymmetry (%) was calculated using the same formula applied for shoulder dynamometry asymmetry analysis, based on the percentage difference between dominant and non-dominant limbs.

### 2.5. Ultrasonographic Evaluation

Ultrasound imaging was performed using a high-resolution B-mode ultrasound device equipped with a linear transducer (7–15 MHz) (Logiq E, GE Healthcare^®^, Waukesha, WI, USA). All measurements were conducted by an experienced physiotherapist (more than 15 years of clinical experience) with participants positioned comfortably and instructed to remain relaxed during image acquisition.

**Figure 3:**
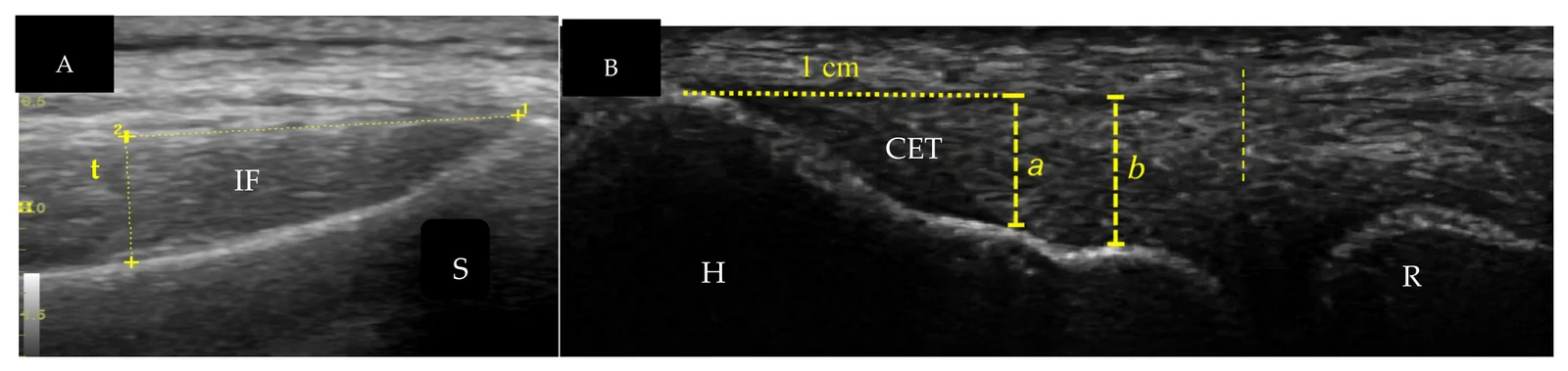
(**A**) Infraspinatus thickness measurement (IF, infraspinatus; S, scapula). Dashed line (t) indicates the measurement of the IF muscle thickness; (**B**) common extensor tendon measurement. H, humerus; R, radius; CET, common extensor tendon. Dashed line (a) indicates tendon thickness measured 1 cm from the top of the lateral epicondyle (ecrb, extensor carpi radialis brevis + edc, extensor digiti communis). Dashed line (b) indicates tendon thickness measured at the plateau ecrb + edc + sup (supinator).

- Infraspinatus Thickness [24]: This variable was evaluated bilaterally under two conditions: repetitions and isometric contraction. Participants were positioned prone with the shoulder in neutral rotation and the arm resting comfortably alongside the body. The transducer was placed transversely over the infraspinatus fossa, inferior to the scapular spine, at the midpoint between the medial scapular border and the posterior acromion. For the repetition condition, measurements were obtained immediately following a standardized repetitive external rotation protocol. For the isometric condition, participants performed a sustained isometric external rotation contraction against manual resistance while ultrasound images were captured. The following variables were analyzed: infraspinatus thickness (pain and non-pain shoulders during repetitions and isometric contractions). Muscle thickness was defined as the perpendicular distance between superficial and deep fascial borders (Figure 3A).
- Common Extensor Tendon Thickness [25,26,27]. This variable was assessed bilaterally at two anatomical regions: 1 cm distal to the lateral epicondyle and plateau region of the tendon insertion. Participants were positioned seated with the elbow flexed to 90°, forearm pronated, and wrist resting comfortably on the examination table. The transducer was aligned longitudinally with the tendon fibers over the lateral epicondyle. For the 1 cm measurement, tendon thickness was quantified 1 cm distal to the tendon insertion at the lateral epicondyle. For the plateau region, thickness was measured at the widest proximal insertional area. The following ultrasound variables were collected: common extensor tendon thickness at 1 cm distal to the lateral epicondyle and at the plateau region in both dominant and non-dominant limbs. Tendon thickness was measured as the maximal anteroposterior diameter in millimeters (Figure 3B).

### 2.6. Statistical Analysis

Statistical analyses were performed using JASP software (JASP 0.98). First, a descriptive analysis of all variables was conducted, with data expressed as mean ± standard deviation (SD) for continuous variables. Data distribution was assessed using the Shapiro–Wilk test. Subsequently, comparisons between the control group and the patient group were performed using the independent samples Student’s *t*-test. In addition, JASP also provided the non-parametric Mann–Whitney U test as a complementary analysis, along with the corresponding effect size; however, for the main presentation of the results, values derived from the *t*-test were reported, including the *p*-value and effect size expressed as Cohen’s d. For additional subgroup comparisons, the same statistical approach was applied, using the independent samples *t*-test and reporting descriptive statistics, *p*-values, and effect sizes. In cases where the parametric test could not be computed due to zero variance following group aggregation, this was explicitly stated in the results tables. The magnitude of the effect size was interpreted using Cohen’s d, with values around 0.2 considered small effects, 0.5 moderate effects, and ≥0.8 large effects. The level of statistical significance was set at *p* < 0.05.

## 3. Results

As shown in Table 2, no significant differences were observed between the control group (CG) and patient group (PG) for most of the isometric strength variables assessed at 0° and 90° of shoulder abduction, including IR/ER peak torque values, IR/ER ratios and interlimb asymmetry indices (*p* > 0.05). Similarly, no statistically significant differences were found in shoulder range of motion variables, total rotational arc, or GIRD values between groups (*p* > 0.05), suggesting comparable global glenohumeral mobility patterns. No significant differences were found between groups in any of the electromyographic ratios analyzed in either limb (*p* > 0.05). Specifically, the IF/PD ratio at 90° of abduction and the UT/LT ratio during shoulder adduction showed comparable values between the experimental and control groups, indicating similar rotator cuff and scapular muscle activation patterns in both populations. Regarding handgrip strength, no significant between-group differences were observed in either dominant or non-dominant limbs, nor in asymmetry indices (*p* > 0.05), indicating preserved distal force production capacity in both populations. However, ultrasound analysis revealed significant between-group differences in infraspinatus muscle thickness. The EG showed greater infraspinatus thickness compared with controls in both pain and non-pain limbs, under both repetitive and isometric conditions (all *p* < 0.05), with large effect sizes (d = from −0.85 to −1.38). These findings suggest greater morphological or adaptive changes in the rotator cuff musculature in subjects with lateral elbow pain. No significant differences were observed in common extensor tendon thickness at any measured level (1 cm and plateau region), indicating the absence of detectable structural differences at the lateral elbow tendon level between groups.

Table 3 shows no significant differences were observed in shoulder dynamometry outcomes at 0° and 90° of abduction (all *p* > 0.05), suggesting preserved isokinetic strength symmetry in both conditions. Regarding shoulder ROM at 90°, no significant differences were observed in ER or total rotational arc (*p* > 0.05). However, IR showed a statistically significant reduction on the pain side compared with the non-pain side (80.3 ± 12.8 vs. 87.1 ± 10.4°, *p* = 0.04), with a moderate effect size (d = −0.58), indicating a mild loss of IR associated with the symptomatic limb. No significant differences were found in electromyographic ratios between sides. The IF/PD ratio at 90° of abduction and the UT/LT ratio during shoulder abduction showed comparable values between limbs (*p* > 0.05), indicating similar neuromuscular activation patterns of the rotator cuff and scapular stabilizers. Handgrip strength did not differ between the pain and non-pain sides (*p* = 0.89), suggesting preserved distal force production capacity regardless of symptomatology. Ultrasound analysis revealed no significant differences in infraspinatus muscle thickness between limbs under repetitive or isometric conditions (*p* > 0.05). In contrast, common extensor tendon thickness showed a tendency toward higher values on the pain side, reaching statistical significance at the plateau region (0.48 ± 0.10 vs. 0.40 ± 0.08, *p* = 0.02), with a moderate effect size (d = 0.68).

## 4. Discussion

The main finding of this study was that unilateral elbow pain in padel players was not associated with widespread deficits in shoulder strength, scapular muscle activation, handgrip strength, or common extensor tendon morphology when compared with asymptomatic players. However, players with elbow pain exhibited significantly greater infraspinatus muscle thickness during both dynamic and isometric tasks, while the within-subject analysis revealed reduced shoulder internal rotation range of motion and increased common extensor tendon thickness on the painful side.

When comparing symptomatic and asymptomatic padel players, the main finding was that the only consistent between-group differences were structural, specifically involving the infraspinatus muscle. Symptomatic players exhibited significantly greater IF thickness (hypertonic muscle) under all ultrasound conditions, including dynamic and isometric tasks, and in both limbs, with large effect sizes (d = 0.85–1.38), suggesting a clinically relevant adaptation. Although no previous studies have specifically examined infraspinatus thickness in overhead athletes with elbow symptoms, structural and functional adaptations of this muscle have been reported in several shoulder disorders [28,29]. Given the role of the infraspinatus in glenohumeral stability and force transmission during overhead activities [30,31], the increased thickness observed in symptomatic players may represent a chronic neuromuscular adaptation aimed at maintaining shoulder stability despite distal elbow symptoms, consistent with the kinetic chain model proposed by Kibler et al. [32].

Interestingly, all participants remained active competitive players despite their symptoms, which may explain the absence of deficits in shoulder strength, mobility, or grip strength, supporting the concept that musculoskeletal pain does not necessarily compromise physical performance [29]. Furthermore, the bilateral increase in infraspinatus thickness suggests that these adaptations are not solely a local response to pain but rather reflect global neuromuscular adaptations of the shoulder stabilizing system. This interpretation is supported by the bilateral demands placed on the rotator cuff during racket sports and by evidence from the cross-education literature demonstrating that unilateral loading can induce bilateral neuromuscular adaptations through central neural mechanisms [33,34,35].

In the comparison between the pain and non-pain side in padel players with lateral elbow pain, a generally symmetrical profile was observed, with preserved shoulder strength, neuromuscular activation, and grip strength. Differences were mainly restricted to a reduction in glenohumeral IR and increased common extensor tendon thickness on the symptomatic side. No significant between-limb differences were found in isokinetic shoulder strength or IR/ER ratios at 0° and 90° of abduction, indicating preserved rotator cuff force-generating capacity. This supports previous evidence suggesting that lateral elbow pain is not necessarily associated with proximal strength deficits, but rather reflects load-related adaptations within the upper-limb kinetic chain [33,36]. Similarly, electromyographic ratios (IF/PD and UT/LT) showed no differences between sides, suggesting preserved scapular and rotator cuff activation patterns. This is consistent with literature indicating that scapular control alterations are often task-specific and may not be detected under isolated or non-sport-specific testing conditions [37]. The only consistent functional alteration was a reduction in IR on the pain side, consistent with GIRD-like adaptations described in overhead athletes. Such deficits have been associated with altered shoulder mechanics and increased distal loading, supporting a kinetic chain mechanism rather than an isolated elbow pathology [38,39]. From a structural perspective, increased thickness of the common extensor tendon on the symptomatic side aligns with the tendon continuum model, where tendon thickening reflects chronic load-induced adaptation and/or degenerative change [30]. In contrast, no relevant differences were observed in infraspinatus thickness, suggesting minimal proximal muscular adaptation. Grip strength remained symmetrical between sides, indicating preserved distal functional capacity despite the presence of symptoms, in agreement with previous findings in lateral elbow tendinopathy [40]. Overall, these findings suggest that lateral elbow pain in padel players was mainly associated with localized tendon adaptations and a specific reduction in shoulder IR, rather than global impairments in strength or neuromuscular control, reinforcing a kinetic chain-based interpretation of the condition.

The practical implication of these findings is that lateral elbow pain in padel players appears to be more closely related to alterations in movement patterns than to deficits in shoulder strength or scapular muscle activation. Specifically, the reduced shoulder IR-ROM observed on the symptomatic side may alter upper-limb kinematics, increasing mechanical stress on the common extensor tendon during repetitive stroke execution. This interpretation is consistent with the movement system approach proposed by Sahrmann et al. [41], which suggests that repeated movement impairments can increase tissue stress and contribute to the development of musculoskeletal disorders. Therefore, clinical assessment and rehabilitation in padel players should emphasize the evaluation and correction of movement impairments, particularly shoulder mobility [37].

### 4.1. Limitation Section

First, the cross-sectional design did not allow causal inferences between shoulder adaptations, tendon morphology, and lateral elbow pain; future longitudinal or prospective studies should address this limitation to clarify temporal and causal relationships. Second, the relatively small and sport-specific sample of padel players limited the generalizability of the findings to other overhead or racquet sport populations; future research should include larger and more diverse cohorts to improve external validity. Finally, the use of isometric and isokinetic assessments in non-sport-specific conditions may not have fully captured functional deficits during dynamic play; future studies should incorporate sport-specific and ecologically valid testing protocols.

### 4.2. Practical Application

These findings suggest that clinical assessment and management of lateral elbow pain in padel players should not be limited to the elbow itself, as proximal shoulder function appears largely preserved. Interventions may benefit from targeting specific deficits in glenohumeral internal rotation and addressing overall upper-limb load management rather than focusing on global strength deficits. The observed increase in common extensor tendon thickness supports the importance of local tendon loading strategies within a progressive rehabilitation model. Overall, a kinetic chain-based approach integrating shoulder mobility and tendon-specific loading appears more appropriate than isolated elbow-focused treatment.

## 5. Conclusions

Comparisons between the control and experimental groups, as well as between pain and non-pain sides, revealed a largely preserved profile of shoulder strength, neuromuscular function, and grip strength in padel players with lateral elbow pain. However, the experimental group showed greater infraspinatus muscle thickness compared with controls, suggesting possible proximal morphological adaptations. The main within-subject differences were a reduction in glenohumeral internal rotation and increased common extensor tendon thickness on the symptomatic side.

## Figures and Tables

**Table 2:** Inter-subject comparison between the patient (PG) and control (CG) groups of all studied variables. Data are presented as mean ± standard deviation (SD).

Variable	CG	PG	*p* Value	Cohen’s d
Shoulder dynamometry at 0°				
Internal rotation (dominant)	11.86 ± 4.06	10.56 ± 5.24	0.45	-
Internal rotation (non-dominant)	10.68 ± 4.52	11.11 ± 6.60	0.83	-
External rotation (dominant)	7.94 ± 3.00	8.03 ± 3.32	0.94	-
External rotation (non-dominant)	8.98 ± 2.96	8.14 ± 2.15	0.38	-
IR/ER ratio (dominant)	0.72 ± 0.33	0.89 ± 0.54	0.29	-
IR/ER ratio (non-dominant)	0.91 ± 0.25	0.91 ± 0.39	0.97	-
IR asymmetry (%)	10.99 ± 17.54	0.62 ± 24.17	0.19	-
ER asymmetry (%)	−12.80 ± 22.87	−3.18 ± 27.28	0.30	-
Shoulder dynamometry at 90°				
Internal rotation (dominant)	13.69 ± 4.87	10.45 ± 3.49	0.05	-
Internal rotation (non-dominant)	12.77 ± 4.35	11.47 ± 3.68	0.39	-
External rotation (dominant)	11.48 ± 4.29	11.21 ± 3.70	0.86	-
External rotation (non-dominant)	11.69 ± 4.55	10.88 ± 4.04	0.61	-
IR/ER ratio (dominant)	0.91 ± 0.42	1.14 ± 0.49	0.18	-
IR/ER ratio (non-dominant)	0.94 ± 0.28	1.01 ± 0.43	0.57	-
IR asymmetry (%)	5.38 ± 15.08	−5.20 ± 29.79	0.23	-
ER asymmetry (%)	−0.82 ± 25.81	4.57 ± 33.83	0.63	-
Shoulder goniometry at 90°				
Dominant IR (°)	74.13 ± 13.01	79.47 ± 12.92	0.27	-
Dominant ER (°)	100.33 ± 12.53	99.13 ± 12.09	0.79	-
Non-dominant IR (°)	85.27 ± 13.05	86.60 ± 9.40	0.75	-
Non-dominant ER (°)	99.60 ± 11.56	99.47 ± 15.06	0.98	-
TROM (dominant)	174.50 ± 14.95	178.60 ± 16.82	0.49	-
TROM (non-dominant)	184.86 ± 19.54	186.07 ± 15.95	0.86	-
GIRD	−11.14 ± 11.41	−7.13 ± 10.11	0.32	-
Electromyography				
IF/PD ratio (dominant, 90° abduction)	0.93 ± 0.29	0.88 ± 0.32	0.61	-
IF/PD ratio (non-dominant, 90° abduction)	0.85 ± 0.33	0.87 ± 0.30	0.87	-
UT/LT ratio (dominant, shoulder abduction)	0.33 ± 0.40	0.29 ± 0.45	0.77	-
UT/LT ratio (non-dominant, shoulder abduction)	0.40 ± 0.38	0.35 ± 0.42	0.68	-
Ultrasound				
Infraspinatus thickness (dominant, repetitions)	0.93 ± 0.28	1.23 ± 0.41	0.03 *	−0.85
Infraspinatus thickness (dominant, isometrics)	1.34 ± 0.30	1.85 ± 0.45	0.01 *	−1.30
Infraspinatus thickness (non-dominant, repetitions)	0.86 ± 0.25	1.17 ± 0.42	0.02 *	−0.89
Infraspinatus thickness (non-dominant, isometrics)	1.35 ± 0.27	1.93 ± 0.48	0.01 *	−1.38
Common extensor tendon thickness (1 cm; dominant)	0.43 ± 0.07	0.45 ± 0.08	0.42	-
Common extensor tendon thickness (plateau region; dominant)	0.48 ± 0.10	0.50 ± 0.11	0.56	-
Common extensor tendon thickness (1 cm; non-dominant)	0.39 ± 0.07	0.41 ± 0.06	0.33	-
Common extensor tendon thickness (plateau region; non-dominant)	0.40 ± 0.08	0.42 ± 0.09	0.41	-
Handgrip strength				
Dominant handgrip	37.2 ± 14.8	39.5 ± 10.5	0.61	-
Non-dominant handgrip	36.8 ± 10.2	36.0 ± 9.1	0.79	-
Asymmetry (%)	85.5 ± 22.4	91.8 ± 6.9	0.28	-

Abbreviations: IR, internal rotation; ER, external rotation; IF, infraspinatus; PD, posterior deltoid; UT, upper trapezius; LT, lower trapezius; TROM, total range of motion; GIRD, glenohumeral internal rotation deficit. * indicates a statistically significant difference.

**Table 3:** Within-subject comparison between the dominant and non-dominant sides of the patient group (PG) for all studied variables. Data are presented as mean ± standard deviation (SD).

Variable	Pain side	Non-Pain Side	*p* Value	Cohen’s d
Shoulder dynamometry at 0°				
Internal rotation	10.8 ± 5.3	11.4 ± 6.3	0.61	-
External rotation	7.9 ± 3.6	8.5 ± 3.9	0.48	-
IR/ER ratio	0.96 ± 0.55	0.95 ± 0.40	0.92	-
Shoulder dynamometry at 90°				
Internal rotation	10.9 ± 3.4	11.5 ± 4.1	0.50	-
External rotation	11.5 ± 3.7	10.8 ± 4.2	0.44	-
IR/ER ratio	1.15 ± 0.53	1.05 ± 0.41	0.29	-
Shoulder goniometry at 90°				
Internal rotation	80.3 ± 12.8	87.1 ± 10.4	0.04 *	−0.58
External rotation	100.7 ± 13.5	97.4 ± 13.8	0.31	-
TROM	180.5 ± 17.2	182.1 ± 15.7	0.60	-
Electromyography				
IF/PD ratio (90° abduction)	0.13 ± 0.34	0.12 ± 0.33	0.89	-
UT/LT ratio (shoulder abduction)	0.33 ± 0.40	0.40 ± 0.38	0.28	-
Ultrasound				
Infraspinatus thickness (repetitions)	0.93 ± 0.28	0.86 ± 0.25	0.18	-
Infraspinatus thickness (isometrics)	1.34 ± 0.30	1.35 ± 0.27	0.92	-
Common extensor tendon thickness (1 cm)	0.43 ± 0.07	0.39 ± 0.07	0.05	-
Common extensor tendon thickness (plateau region)	0.48 ± 0.10	0.40 ± 0.08	0.02 *	0.68
Handgrip strength				
Handgrip strength	37.2 ± 14.8	36.8 ± 10.2	0.89	-

Abbreviations: TROM, total range of motion. * indicates a statistically significant difference.

## Data Availability

The data presented in this study are available on request from the corresponding author. The data are not publicly available due to ethical condition.

## Abbreviations

The following abbreviations are used in this manuscript:

LEP	lateral elbow pain
LET	lateral elbow tendinopathy
IR	internal rotation
ER	rotation
VAS	visual analog scale
ROM	range of motion
TROM	total of range of motion
GIRD	glenohumeral internal rotation deficit
IF	infraspinatus
PD	posterior deltoid
UT	upper trapezius
LT	lower trapezius
CG	control group
PG	patients group
